# Clinical characteristics and survival analysis in critical and non-critical patients with COVID-19 in Wuhan, China: a single-center retrospective case control study

**DOI:** 10.1038/s41598-020-74465-3

**Published:** 2020-10-16

**Authors:** Ran Tian, Wei Wu, Chunyao Wang, Haiyu Pang, Zhiyu Zhang, Haopeng Xu, Qingfeng Luo, Peng Gao, Jihua Shi, Wenbin Li, Hao Qian, Fan Guo, Taisheng Li, Zhengyin Liu, Jinglan Wang, Xiang Zhou, Yan Qin, Xiaowei Yan, Shuyang Zhang

**Affiliations:** 1 Department of Cardiology, Peking Union Medical College Hospital, Chinese Academy of Medical Sciences & Peking Union Medical College, Beijing, China; 2 Department of Medical Intensive Care Medicine, Peking Union Medical College Hospital, Chinese Academy of Medical Sciences & Peking Union Medical College, Beijing, China; 3 Medical Research Center, Peking Union Medical College Hospital, Chinese Academy of Medical Sciences & Peking Union Medical College, Beijing, China; 4 Chinese Academy of Medical Sciences & Peking Union Medical College, Beijing, China; 5 Department of Gastroenterology, Beijing Hospital, National Center of Gerontology, Institute of Geriatric Medicine, Chinese Academy of Medical Sciences, Beijing, China; 6 Department of Infective Disease, Peking Union Medical College Hospital, Chinese Academy of Medical Sciences & Peking Union Medical College, Beijing, China; 7 Department of Pulmonary and Critical Care Medicine, Peking Union Medical College Hospital, Chinese Academy of Medical Sciences & Peking Union Medical College, Beijing, China; 8 Department of Intensive Care Medicine, Peking Union Medical College Hospital, Chinese Academy of Medical Sciences & Peking Union Medical College, Beijing, China; 9 Department of Nephrology, Peking Union Medical College Hospital, Chinese Academy of Medical Sciences & Peking Union Medical College, Beijing, China

**Keywords:** Infectious diseases, Respiratory tract diseases

## Abstract

Since the outbreak of COVID-19 in China at the end of 2019, the world has experienced a large-scale epidemic caused by the SARS-CoV-2. The epidemiological and clinical course of COVID-19 patients has been reported, but there have been few analyses about the characteristics, predictive risk factors, and outcomes of critical patients. In this single-center retrospective case–control study, 90 adult inpatients hospitalized at Tongji Hospital (Wuhan, China) were included. Demographic, clinical, laboratory tests, and treatment data were obtained and compared between critical and non-critical patients. We found that compared with non-critical patients, the critical patients had higher SOFA score and qSOFA scores. Critical patients had lower lymphocyte and platelet count, elevated D-dimer, decreased fibrinogen, and elevated high-sensitivity C-reactive protein (hsCRP), and interleukin-6(IL-6). More critical patients received treatment including antibiotics, anticoagulation, corticosteroid, and oxygen therapy than non-critical ones. Multivariable regression showed higher qSOFA score and elevation of IL-6 were related to critical patients. Antibiotic usage and anticoagulation were associated with decreased in-hospital mortality. And critical grouping contributed greatly to in-hospital death. Critical COVID-19 patients have a more severe clinical course. qSOFA score and elevation of IL-6 are risk factors for critical condition. Non-critical grouping, positive antibiotic application, and anticoagulation may be beneficial for patient survival.

## Introduction

Since the outbreak of COVID-19 in Wuhan, Hubei at the end of 2019, China has experienced another large-scale epidemic disease caused by a coronavirus after Severe Acute Respiratory Syndrome (SARS). By the end of August 24, 2020, the number of confirmed cases in China exceeded 8, 4981, with more than 4600 deaths^1^. This disease has also spread to 216 countries, areas or territories, with a total of 23,311,719 infected cases and 806,410 confirmed deaths^2^. The World Health Organization (WHO) has claimed COVID-19 as a global pandemic^3^. And health systems in all countries are currently faced with serious challenges.

Although we have acquired a deeper understanding of the disease through autopsy and virological study, no specific treatment seems to be definitive and effective for preventing the disease progression and death of critical patients. According to the diagnosis and treatment guidelines released by China Health and Medical Commission^4^, patients can be divided into four types clinically: mild, ordinary, severe and critical. The clinical manifestation and required treatment vary greatly between different types. Mild and ordinary patients need only supportive treatment, and self-healing cases have been reported, but most of the critically ill patients need mechanical ventilation or even extra-corporeal membrane oxygenation (ECMO) and continuous renal replacement therapy (CRRT). The mortality rate reported in different literatures is between 1–4%^5–7^, but the severity of the illness and the mortality rate can be underestimated due to a large number of asymptomatic infections and mild patients. Most current researches are descriptive studies on patients admitted to hospitals, yet there have been few analyses about the outcomes and risk factors of critical patients until now.

Here, we present the clinical course and outcomes of a group of critical patients with COVID-19, and attempt to identify risk factors for disease progression and in-hospital mortality in these patients. We aim to find some predictive risk factors for early warning, to provide opportunities for timely medical intervention by simple and effective assessment.

## Results

**Figure 1:**
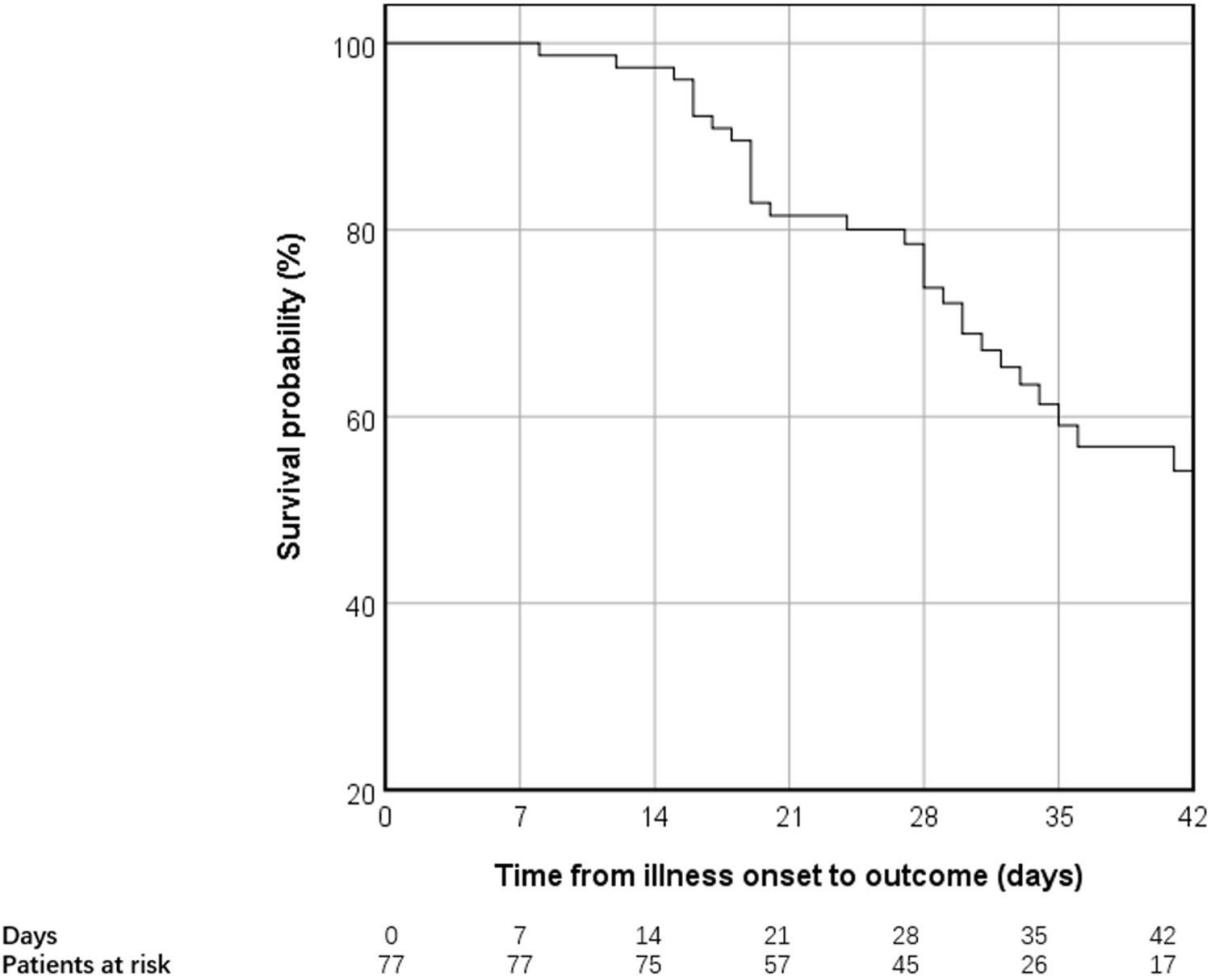
Kaplan–Meier survival analysis of all the patients.

We collected 90 inpatients diagnosed with COVID-19 at the Tongji Hospital (Wuhan, China) from Jan 28th to Feb 28th. Of all the patients, 45 are critical patients and 45 are non-critical patients. All patients were discharged or died before the date of data collection. 32 of the 90 patients died and 48 were discharged. (Fig. 1) The median age of all the patients was 64 years (56–70), ranging from 26 to 92. 48 patients were males and 42 were females. No significant differences were observed between the two groups in terms of age, gender, and comorbidities. (Table 1).

**Table 1:** Demographic and clinical characteristics of patients on admission.

	Total (n = 90)	Non-critical (n = 45)	Critical (n = 45)	p-value
Age, years	64 (56–70)	63 (59–70)	64 (56–71)	0.984
Sex				0.398
Male	48 (53%)	22 (49%)	26 (58%)	
Female	42 (47%)	23 (51%)	19 (42%)	
**Comorbidity**				
Cardiovascular disease	11 (12%)	4 (9%)	7 (15.6%)	0.334
Hypertension	38 (42%)	19 (42%)	19 (42%)	1.000
Diabetes mellitus	17 (19%)	9 (20%)	8 (18%)	0.788
Chronic obstructive pulmonary disease	4 (4%)	2 (4%)	2 (4%)	1.000
Chronic kidney disease	1 (1%)	0 (0%)	1 (1%)	1.000
Cerebrovascular disease	6 (7%)	2 (4%)	4 (9%)	0.677
Malignance	10 (11%)	8 (18%)	2 (4%)	0.044
**Disease progression and outcome**				
Time between illness onset and hospital admission	14 (7–22)	12 (5–15)	16 (10–25)	0.004
Time between hospital admission and outcome	16 (9–26)	18 (13–24)	12 (5–29)	0.455
Time between illness onset to outcome^#^	31 (21–42)	30 (22–34)	32 (19–49)	0.422
Death	32 (36%)	2 (4%)	30 (67%)	< 0.001
**Vital signs**				
HR*, min-1	102 (19)	93 (15)	110 (19)	< 0.001
RR, min-1	20 (20–28)	20 (18–20)	26 (22–35)	< 0.001
SBP*, mmHg	130 (20)	132 (19)	128 (21)	0.288
DBP*, mmHg	80 (13)	82 (13)	78 (13)	0.172
SOFA	3 (1–5)	1 (1–2)	5 (4–7)	< 0.001
qSOFA	1 (0–2)	0 (0–0)	2 (1–2)	< 0.001
**Symptoms**^†^				
Fever	34 (38%)	11 (24%)	23 (40%)	0.009
Cough	50 (57%)	25 (57%)	25 (58%)	0.807
Dyspnea	27 (31%)	4 (9%)	23 (54%)	< 0.001
Expectoration	32 (36%)	11 (24%)	21 (49%)	0.017
Fatigue	44 (50%)	24 (53%)	20 (47%)	0.522

Data are median (IQR), average (SD) or n (%). P values comparing patients are from Student’s t test, chi-square test, or Fisher’s exact test.

*HR* heart rate, *RR* respiratory rate, *SBP* systolic blood pressure, *DBP* diastolic blood pressure, *SOFA* Sequential Organ Failure Assessment score, *qSOFA* quick Sequential Organ Failure Assessment Score.

*The data is normally distributed, thus average (SD) are used.

^**#**^Limited sample number: non-critical 33 samples, critical: 44 samples.

^†^Limited sample number: critical 43 samples.

Vital signs at the time of admission were analyzed. The critical patients had faster heart rate (102 ± 19, per min) and respiratory rate (26, 22–35, per min), higher SOFA score (5, 4–7) and qSOFA score(2, 1–2). More patients in the critical group had symptoms of fever, expectoration, and dyspnea, but most of the symptoms like cough, fatigue, chill, etc. were non-specific and showed no significance between groups (not all shown in the table).

The mortality rate in the critical group was 67%, which was significantly higher than the non-critical group. The median time from onset to admission in the critical group was 16 days (10–25), longer than that in the non-critical group. There was no significant difference in the time of hospitalization and the entire course of disease between the two groups.

Results of blood test at admission for all patients were obtained and analyzed (Table 2.). It was found that the critical group had higher white blood cell (10.25, 7.96–15.14, × 10^9^/L) and neutrophil count (9.21, 6.77–13.10, × 10^9^/L) and lower lymphocyte count. Platelet counts are also lower. Elevated alanine aminotransferase (ALT, 29.0, 20.0–45.5, U/L), total bilirubin (12.2, 7.8–18.9, μmol/L), hypoalbuminemia (29.6, 5.4, g/L), and hyperureaemia (7.6, 5.6–12.7) were also more commonly observed in the critical group. Meanwhile, critical patients are more likely to show prolonged partial thromboplastin time (PT, 16.7, 15.1–18.2, s), increased D-dimer, and decreased fibrinogen. In terms of inflammatory factors, the proportion of patients with elevated ferritin and elevated hsCRP and IL-6 in the critical group was also larger.

**Table 2:** Laboratory test results and treatment.

	Total(n = 90)	Non-Critical(n = 45)	Critical(n = 45)	p
White blood cell, × 10^9^/L	7.56(5.08–10.55)	5.90(3.97–7.25)	10.25(7.96–15.14)	< 0.001
Neutrophil, × 10^9^/L	6.04(3.36–9.46)	3.72(2.40–5.49)	9.21(6.77–13.10)	< 0.001
Lymphocyte, × 10^9^/L	0.84(0.48–1.22)	1.04(0.80–1.43)	0.54(0.33–0.89)	< 0.001
Hemoglobin, g/L	120.5(105.8–132.3)	119(107–132)	124(105–136)	0.470
Platelet, × 10^12^/L	192.50(136.25–285.25)	230.00(164.00–310.50)	159.00(102.00–235.50)	0.005
ALT, U/L	24.5(14.0–45.0)	19.0(11.0–38.5)	29.0(20.0–45.5)	0.043
Total bilirubin, μmol/L	10.5(7.3–15.8)	9.9(6.8–12.8)	12.2(7.8–18.9)	0.047
Serum creatine, μmol/L	70.0(56.5–91.3)	64.0(57.5–80.5)	79.0(50.5–106)	0.211
BUN, mmol/L	5.1(3.9–8.1)	4.1(3.4–4.9)	7.6(5.6–12.7)	< 0.001
Albumin*, g/L	31.7(5.4)	33.8(4.6)	29.6(5.4)	< 0.001
INR	1.1(1.0–1.4)	1.0(1.0–1.1)	1.3(1.2–1.5)	< 0.001
PT, s	14.5(13.7–17.1)	13.7(13.4–14.3)	16.7(15.1–18.2)	< 0.001
APTT, s	39.4(35.7–44.4)	38.7(35.7–45.1)	40.8(35.5–43.7)	0.704
D-dimer > 1 mg/L	66(73%)	25(56%)	41(91%)	< 0.001
Fibrinogen, g/L	4.7(3.3–6.0)	5.25(4.1–6.2)	3.9(2.6–5.4)	0.005
Fer > 400 ug/L	77(86%)	33(73.3%)	44(97.8%)	0.001
hsCRP > 3 mg/L	77(86%)	34(76%)	43(96%)	0.007
IL-6 > 14 pg/mL #	50(56.2%)	13(29%)	37(84%)	< 0.001
**Treatment**				
Antibiotics	47(52%)	16(36%)	31(69%)	0.002
Antiviral	49(54%)	21(47%)	28(62%)	0.138
Anticoagulation	25(28%)	0(0%)	25(56%)	< 0.001
Corticosteroid	43(48%)	9(20%)	34(76%)	< 0.001
IVIG	38(42%)	3(7%)	35(78%)	< 0.001
Anti-IL6 therapy	6(7%)	1(2%)	5(11%)	0.203
CRRT	7(8%)	0(0%)	7(16%)	0.012
High flow oxygen	15(17%)	1(2%)	14(31%)	< 0.001
Prone ventilation	14(16%)	1(2%)	13(16%)	< 0.001
BiPAP	11(12%)	2(4%)	9(20%)	0.024
Invasive ventilation	36(40%)	1(2%)	35(78%)	< 0.001
ECMO	4(4%)	0(0%)	4(9%)	0.117

Data are median (IQR), average (SD) or n (%). P values comparing patients are from Student’s t test, chi-square test, or Fisher’s exact test.

*ALT* alanine aminotransferase, *BUN* blood urea nitrogen, *INR* international normalized ratio, *PT* partial thromboplastin time, *APTT* activated partial thromboplastin time. *IVIG* intravenous immunoglobin, *ECMO* Extracorporeal membrane oxygenation.

*The data is normally distributed, thus average (SD) are used.

More than two-thirds of the 90 patients (n = 62) received varying level of oxygen therapy support, of which 36 patients had mechanical ventilation. The critical group has higher requirements for oxygen therapy support. More patients need high-flow oxygen inhalation, prone ventilation, BiPAP and mechanical ventilation. The usage of antiviral drugs is similar between the two groups. More patients were treated with antibiotics, intravenous immunoglobin (IVIG), and glucocorticoid in the critical group; more critical patients received treatment for complications, including renal replacement therapy and anticoagulation. 6 of the patients received anti-IL-6 treatment (5 died) and 4 received ECMO (3 died).

To find the possible indicators for patients’ group, potential influential factors including differences between the two groups and factors related to clinical diagnosis and treatment were screened. Age, gender, qSOFA scores, low lymphocyte (less than 0.8), high D-dimers (greater than 1), and high IL-6 (greater than twice the upper limit) were analyzed in conditional logistic regression using COX survival analysis. We found that qSOFA scores and increased IL-6 were significantly associated with critical group (Table 3).

**Table 3:** Risk factors associated with critical patients.

	OR	95% CI	p
Sex	1.03	0.22–4.86	0.969
Age	0.95	0.89–1.01	0.950
qSOFA	12.69	3.5–46.2	** < 0.001**
LY < 0.8	4.30	0.84–22.08	0.081
D-dimer > 1 mg/L*	1.56	0.26–0.93	0.624
IL-6 > 14 pg/mL*	6.67	1.53–29.05	**0.012**

P values are from logistic regression.

*OR* odds ratio, *qSOFA* quick Sequential Organ Failure Assessment Score, *LY* lymphocyte, *IL-6* interleukin-6.

*Normal range: D-Dimer < 0.5 mg/L, IL-6 < 7 pg/mL.

**Figure 2:**
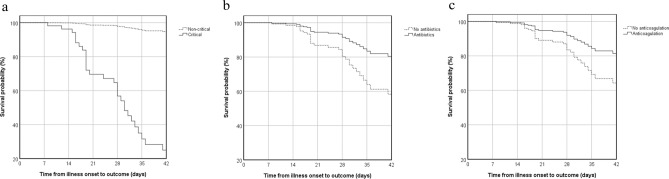
Cox regression survival analysis of all patients with COVID-19. (**a**) Comparison between critical and non-critical group. (**b**) Comparison between antibiotics with no antibiotics usage. (**c**) Comparison between anticoagulation with no anticoagulation usage.

Log rank tests were performed on single risk factors on patient outcome. Many factors show correlation to the outcome, including gender, disease grouping, baseline heart rate and respiratory rate, SOFA and qSOFA scores, lymphocyte counts, platelet count, liver and renal function, coagulation, several inflammatory factors, glucocorticoid usage, BiPAP, mechanical ventilation, etc. (data not shown). Corticosteroid was the first reported drug that can lower 28-day mortality of patients hospitalized with COVID-19 among those who were receiving either invasive mechanical ventilation or oxygen alone at randomization but not among those receiving no respiratory support^8^, which supported our findings that corticosteroid (p = 0.016), non-invasive ventilation (p = 0.014) and invasive ventilation (p < 0.001) were considered to be linked with in-hospital mortality. Then, five factors, especially treatment factors, were selected in multivariate regression analysis, among which age, disease grouping, antibiotics and anticoagulation were related to death. (Fig. 2. and Table 4.)

**Table 4:** Risk factors associated with in-hospital mortality.

	Univariate survival analysis	Multivariate survival analysis	
	p-value	HR (95% CI)	p-value
Age	–	1.05 (1.02–1.09)	**0.002**
**Sex**			
Male	**0.039**	2.01 (0.935–4.334)	0.074
Female	**Referee**		
Critical/non-critical	** < 0.001**	25.70 (5.51–119.92)	** < 0.001**
Antibiotics	0.572	0.405 (0.18–0.91)	**0.029**
Antiviral	0.341		
Anticoagulation	0.226	0.465 (0.22–0.99)	**0.048**
Corticosteroid	**0.016**		
IVIG	0.208		
Anti-IL6 therapy	0.148		
CRRT	0.285		
High flow oxygen	0.313		
Non-invasive ventilation	**0.014**		
Invasive ventilation	** < 0.001**		
ECMO	0.326		
Prone ventilation	0.490		

Note: for univariant, P values are from Log rank test, for multivariant, P values are from Cox regression.

*HR* hazard ratio, *CI* confidence interval, *IVIG* intravenous immunoglobin, *CRRT* continuous renal replacement therapy, *ECMO* extracorporeal membrane oxygenation.

## Discussion

COVID-19 has now become a global pandemic, and medical systems in many countries are facing serious challenges. Survival of critical and non-critical patients are significantly different, so the early identification of critical patients is very important. This retrospective research shows that critical patients have more severe clinical situation and worse prognosis, which requires medical support of high grade, including higher level of oxygen therapy, supportive therapy and organ replacement therapy. As seen in other literatures, the risk of complications is also higher in critical patients, including respiratory failure, acute respiratory distress syndrome (ARDS), secondary infections, myocardial injury, liver and kidney dysfunction, etc.^9,10^. This is consistent with our observations during clinical process.

There are several clinical manifestations worth mentioning. For example, dyspnea is not the most significant symptom of COVID-19, but it is relatively prominent in critical patients, so it may be a suggestive clue. Critical patients have significantly lower lymphocytes compared with non-critical ones, which is consistent with autopsy results^11^, indicating a more severe bone marrow suppression and lymphocyte failure^12^. In addition, worse coagulation function is more likely to occur in critical patients, and the disseminated intravascular coagulation (DIC) indicators like platelet count, PT, and D-dimer have changed significantly. Acrotic gangrene was observed in several patients (not shown in the data), indicating a potential DIC. By comparing the pathogenesis of SARS, MERS, and other viral pneumonias, we find that some critical patients may need low-molecular-weight heparin anticoagulant therapy^13^. During our clinical practice, the gangrene in critical patients did significantly improve after low-molecular-weight heparin treatment. Also, data analysis also provided supportive evidence that anticoagulation may be beneficial for patient outcomes.

In addition to the risk factors like elder age that have been reported^9^, we have found that high qSOFA scores and IL-6 elevation may help to identify critical patients. The qSOFA score can make quick evaluation of patient condition based on vital signs and consciousness^14^. Although previous research revealed that qSOFA score may have limited utility for predicting mortality in an ICU setting compared with SOFA score or SIRS criteria^15^, we believe that its convenience may have great value for practical application in current situation especially for primary medical institutions and emergency with insufficient medical resources. The elevation in IL-6 suggests the possible role of cytokine storm in the progression of COVID-19 and potential therapeutic targets^16^.

Treatment is another field we paid attention to, although no specific treatment has been proven effective. We mainly provided supportive treatment according to clinical symptoms. The use of more antibiotics in critical patients, combined with relatively high neutrophil count, indicates more possibility of secondary opportunistic or drug-resistant infections during the long period of disease and mechanical ventilation. Pathological examination also confirmed that bacterial infection is an important pathological process which may aggravate alveolar injury and ventilation dysfunction among dead patients. Multivariate regression analysis showed that positive antibiotic usage may be beneficial for patient outcomes. Glucocorticoid is another controversy. Although it can be used as an anti-shock and anti-inflammatory agent, the use of glucocorticoid may contribute to infection. The experience of SARS has deepened our understanding of the role of glucocorticoid in severe viral pneumonia^17^, but the balance between the suppressive effects of glucocorticoid on immunity and the positive effects of its anti-inflammatory role requires further research.

This study will provide possible supportive evidence for potential treatments by comparing effect made by different treatments on patients’ survival curves. Antiviral drugs, anti-IL-6 therapy and proper glucocorticoid usage may all have potential therapeutic effects^18^, as many clinical trials are still ongoing. We are urgently expecting some promising results, which is of vital importance in clinical course.

Our study has some limitations. First, only 90 patients with confirmed COVID-19 were included; the suspicious and undiagnosed cases were excluded in the analyses. It would be better to absorb more patients to gain a comprehensive understanding of 2019­nCoV. Second, the lack of availability to some medical records limited our analyses of certain data. Some blood tests have not been performed in all patients for realistic reasons. In addition, since some of the critical patients were transferred to our hospital in urgent need for medical support due to the outbreak of COVID-19, it is difficult to evaluate the effect of previous treatment, and may lead to some unknown bias to sample selection. Moreover, this study is a retrospective case–control design of a single center and a rather small sample size may limit our selection of potential risk factors in the multiple regression analysis to some degree. The possibility of selection bias may exist and the results need careful interpretation.

In conclusion, this study compared the clinical characteristics between critical and non-critical COVID-19 inpatients, and qSOFA score and elevation of IL-6 are risk factors for critical condition. In multivariate survival analysis, the treatment of antibiotics and anticoagulation were significant factors for in-hospital mortality.

## Methods

### Study design and participants

This study is a retrospective case–control study, including adult (≥ 18 years old) inpatients hospitalized at Tongji Hospital (Wuhan, China) from Jan 9th to Feb 28th. All adult patients who were diagnosed with COVID-19 according to WHO interim guidance were screened. All patients were discharged or died before the date of data collection. These patients were divided into critical and non-critical group which include mild, ordinary and severe patients. The diagnosis was made according to the diagnosis and treatment guidelines (7th ed) released by China Health and Medical Commission. Mild patients are defined as patients with minor clinical symptoms and no imaging manifestations. Ordinary patients are defined as patients with typical clinical and imaging manifestations. Severe patients are defined as patients meeting at least one of the following criteria: respiratory rate over 30/min, SpO2 less than 93% at rest, PaO2/FiO2 less than 300 mmHg, rapidly progressive lung imaging lesions. Critical patients are defined as patients who need mechanical ventilation or shock or have other organ failure.

The criteria for discharge were absence of fever for at least 3 days, substantial improvement in both lungs in chest CT, clinical remission of respiratory symptoms, and two throat-swab samples negative for SARS-CoV-2 RNA obtained at least 24 h apart.

Personal identifiable information was removed from all cases during the study to protect privacy. The study was approved by the Research Ethics Commission of Tongji Hospital (Wuhan, China) and the requirement for informed consent was waived by the Ethics Commission. All methods were performed in accordance with the relevant guidelines and regulations.

### Data collection

Epidemiological, demographic, clinical, laboratory, treatment, and outcome data were extracted from electronic medical records in the hospital form by two physicians independently using a standardized collection. The data consistency and accuracy were checked by a third researcher.

### Laboratory procedures

Methods for laboratory confirmation of COVID-19 have been described elsewhere. According to the latest guideline, COVID-19 can be diagnosed by either serum antibody or nucleic acid detection. The SARS-CoV-2 RNA was detected by Centers for Disease Control and Prevention, and the detection of serum antibody was done by local health institutions.

Routine blood examinations were complete blood count, coagulation profile, serum biochemical tests (including renal and liver function, creatine kinase, lactate dehydrogenase, and electrolytes), myocardial enzymes, inflammatory factors (including high-sensitivity C-reactive protein (hsCRP), interleukin-1 (IL-1), interleukin-2 (IL-2), interleukin-6 (IL-6), interleukin-8 (IL-8), interleukin-10(IL-10) tumor necrosis factor α (TNF α) and serum ferritin), and procalcitonin. Baseline examination data were obtained for all patients. Frequency of examinations was determined by the treating physician according to disease progression.

### Statistical analysis

The continuous data were expressed by mean (SD) or median (IQR) depending on whether they are normally distributed. They were tested by t-test or Mann-Whitey U test depending on normal distribution and homogeneity in variance. The Shapiro–Wilk test was used to test whether the continuous data were normally distributed. Categorical data were expressed by number (percentage), tested by Chi-square or Fisher’s exact test. Multivariant analysis used logistic regression. For survival analysis, univariant analysis was Kaplan–Meier analysis, and multivariant analysis was COX regression. The significance was defined as p value below 0.05. All the data analysis was conducted in SPSS 21.0(IBM Corporation, Armonk, NY, USA).

## Data Availability

The datasets used and/or analyzed during the current study are available from the corresponding author on reasonable request.
